# Women with COPD by biomass show different serum profile of adipokines, incretins, and peptide hormones than smokers

**DOI:** 10.1186/s12931-018-0943-4

**Published:** 2018-12-04

**Authors:** Oliver Pérez-Bautista, Martha Montaño, Rogelio Pérez-Padilla, Joaquín Zúñiga-Ramos, Mariana Camacho-Priego, Tonatiuh Barrientos-Gutiérrez, Ivette Buendía-Roldan, Yadira Velasco-Torres, Carlos Ramos

**Affiliations:** 1 Departamento de Investigación en Tabaquismo y EPOC, Instituto Nacional de Enfermedades Respiratorias Ismael Cosío Villegas (INERICV), Ciudad de México, Mexico; 2 Departamento de Investigación en Fibrosis Pulmonar, Instituto Nacional de Enfermedades Respiratorias Ismael Cosío Villegas (INERICV), Talpan 4502, C.P. 14080 Ciudad de México, Mexico; 3 Laboratorio de Inmunobiología y Genética, Instituto Nacional de Enfermedades Respiratorias Ismael Cosío Villegas (INERICV), Ciudad de México, Mexico; 4 Centro de Investigación en Salud Poblacional, Instituto Nacional de Salud Pública (INSP), Cuernavaca, Morelos Mexico; 5 Doctorado en Ciencias Biológicas y de la Salud, Universidad Autónoma Metropolitana-Xochimilco (UAMX), Ciudad de México, Mexico

**Keywords:** Adipokines, Biomass smoke exposure, COPD, Incretins, Insulin, Peptide hormones, Tobacco smoking

## Abstract

**Background:**

The main causes of COPD are tobacco smoking (COPD-TS) and biomass smoke exposure (COPD-BS). COPD-TS is known to induce changes in adipokines, incretins, and peptide hormones, frequent biomarkers of inflammation; however, it is unknown if similar changes occur in COPD-BS.

**Methods:**

Clinical and physiological characteristics, and serum concentration of C-peptide, ghrelin, GIP, GLP-1, glucagon, insulin, leptin, PAI-1, resistin, and visfatin were measured in women with COPD-BS, COPD-TS, and healthy controls.

Data were compared with one-way ANOVA and Tukey’s post hoc test; nonparametric were expressed as median (interquartile ranges), with Kruskal-Wallis and Dunn’s post-hoc test. Multivariate analysis, age, BMI, MS, and FEV_1_% pred with levels of inflammatory mediators in COPD women.

**Results:**

FEV1% pred, FVC% pred, and FEV1/FVC ratio were decremented in COPD. In COPD-TS increased C-peptide, ghrelin, GIP, GLP-1, and leptin, and reduced glucagon, PAI-1, resistin, and visfatin. In COPD-BS enlarged ghrelin, insulin, leptin, and PAI-1 comparatively with COPD-TS and control, while C-peptide and GLP-1 relatively with controls; conversely, glucagon, and resistin were reduced. Multivariate analysis showed association of ghrelin, insulin, PAI-1, and visfatin with BS exposure.

**Conclusions:**

women with COPD-BS have a distinct profile of adipokines, incretins, and peptide hormones, and specifically with ghrelin, insulin, PAI-1, and visfatin related to BS exposure.

## Background

Chronic obstructive pulmonary disease (COPD) is an important cause of global mortality. Tobacco smoking (TS) is the primary cause of COPD, yet, chronic exposure to biomass smoke (BS), mainly wood smoke, is the second risk factor [1]. The clinical profile of COPD associated with BS (COPD-BS) exposure, and its prognostic factors were recently described [2]. COPD-BS has remarkable pathophysiological differences compared to COPD associated with tobacco smoking (COPD-TS) [3, 4]. Patients with COPD-BS are commonly women, older than 65 years, shorter compared with patients with COPD-TS [2–4]. It is pertinent notes that in Mexico women tend to be more exposed to BS than men, due to them prepared the meals, as a domestic role. In pathophysiological studies, women with COPD-BS experience airway disease, with persistent cough, dyspnea, phlegm, airway thickening, air trapping, and *cor pulmonale* in more advanced stages [5–7], yet, they rarely present severe airflow obstruction, low diffusing capacity or lung emphysema, which are common features of COPD-TS [8]. Given these differences, it is reasonable to assume that COPD-BS and COPD-TS could express different patterns of metabolic biomarkers.

Type 2 diabetes mellitus (T2DM) and hyperinsulinemia are frequent COPD comorbidities. Pre-existing and incident T2DM have been associated with worse outcomes in COPD-TS [9].

Adipokines play an important role in the development of metabolic pathologies, such as metabolic syndrome (MS) and T2DM [10, 11]; thus monitoring adipokines in serum and BAL fluid could be useful to predict metabolic complications in patients with COPD [12].

Varieties of metabolic and inflammatory mediators of T2DM, as well as dysregulation of adipokines due to a metabolically active adipose tissue, are associated with COPD-TS [9, 13]; however, it is not known if the same pattern occurs in COPD-BS.

*We hypothesized COPD-BS represent a metabolically different phenotype to COPD-TS. Consequently, we focus our analysis in compare the concentration of adipokines, incretins, and peptide hormones in COPD-BS, COPD-TS, and in a healthy women control group; in addition, a multivariate analysis was perform to associate age, BMI, MS, and FEV1% pred with the level of inflammatory mediators in COPD-BS.*

## Methods

### Study population

Women with clinical and functional diagnosis of COPD were recruited at the COPD clinic of the Instituto Nacional de Enfermedades Respiratorias Ismael Cosío Villegas (INERICV) in Mexico City, from May 2014 to May 2017. The diagnosis of COPD was established by post-bronchodilator pulmonary function tests, including the percent predicted forced expiratory volume in the 1st second (FEV_1_% pred), percent predicted FVC (FVC% pred), and FEV_1_/FVC < 70% [1]. COPD was classified according to exposure histories; we recruited half of the women with BS exposure, and the remaining with TS exposure. A group of healthy females (*n* = 70) without history of TS or BS or any other disease were recruited as the control group.

Demographic, anthropometric, and clinical data were collected, including the history of TS (> 10 pack-years), cumulative exposure to BS was expressed as hour-years, and average hours/day multiplied by the number of years, by clinical interview and a standardized Spanish version of the American Thoracic Society questionnaire [1, 14, 15]; supplemented by additional questions directly related to cooking fuels. None of the COPD women reported exposure to BS or TS over the last 10 years. Wood was the fuel used for cooking in all COPD-BS; none was a smoker. We excluded subjects with a history of other chronic pulmonary conditions such as asthma, tuberculosis or bronchiectasis, or any other non-pulmonary disease. None of the controls or patients recruited reported cardiovascular CV diseases or T2DM. None of the women with COPD was exposed to BS or TS the last 10 years.

Subjects with COPD-BS were matched one-to-one with subjects with COPD-TS by the FEV_1_% pred. Patients with COPD were clinically stable and without exacerbation for at least 6 weeks prior to the study. We defined a control group which included women with no COPD and without exposure to either tobacco smoke or biomass, to provide a group to which both COPD-BS and COPD-TS could be compared too.

### Pulmonary function tests

Pre- and post-bronchodilation spirometry was performed in all women following the procedures recommended by the American Thoracic Society/European Respiratory Society [1], with a dry rolling-seal volume spirometer (Sensormedics, Yorbalinda, CA), and using Mexican standard reference equations [15, 16].

### Blood samples

Serum was obtained from whole blood in all women; samples were centrifuged (5000×g, 4 °C) in tubes without EDTA. Serum was kept at − 20 °C until analysis. Adipokines were measured following standard procedures at the hospital, which included morning only bleeding with at least 8 h of fasting.

### Inflammatory biomarkers measure

C-peptide, ghrelin, GIP, GLP-1, glucagon, insulin, leptin, PAI-1, resistin, and visfatin were measured with the commercial kit Bio-Plex Pro™, Human Diabetes Assays (Bio-Rad Laboratories Inc., Hercules, CA, USA) according the specifications of fabricant. Concentrations were expressed as median (interquartile ranges) of ng/ml in serum.

### Statistical analysis

Continuous demographic and clinical data were expressed as mean and standard deviation, and groups were compared with a one-way ANOVA and Tukey’s post hoc test. Concentrations of biomarkers of T2DM (nonparametric data) were expressed as median (interquartile ranges), and were analyzed with Kruskal-Wallis followed by Dunn’s post-hoc test.

Data of serum concentration were expressed as ng/ml or Ln (logarithmic natural) function of ng/ml. GraphPad Prism (v 6.1 GraphPad Software, Inc., San Diego, CA, USA).

Multivariate analysis was performing to associate age, BMI, MS, and FEV_1_% pred, using the XLSTAT 2016.5 Addinsoft; a *P < 0.05* was considered statistically significant.

## Results

### Patient characteristics

Clinical data from healthy and COPD subjects are shown in Table 1. Women in the COPD-BS group were older than those in the COPD-TS and control groups. Women in the COPD-TS group were taller than women in the COPD-BS and control groups. Mean exposure to BS was 361 ± 177 h-years, and 7.17 ± 2.56 h-days; whereas in TS was a mean cumulative consumption of tobacco of 37.28 ± 11.43 pack-years compared to normal participants. COPD patients had a lower FEV_1_% pred, FVC% pred and FEV_1_/FVC ratio; no significant differences between COPD groups were observed. C-reactive protein (CRP) was elevated in both COPD groups compared to controls; serum glucose was higher in COPD-BS compared with COPD-TS and controls (Table 1). From the total of women with COPD-BS, 48 were in GOLD stage I-II, 22 in GOLD-stage III-IV; while from women with COPD-TS 32 were in GOLD-stage I-II and 38 in stage GOLD stages III-IV.

**Table 1:** Demographics, physiological and clinical characteristics in women with and without COPD associated with TS and BS, and control healthy women. *n* = 70

	C	COPD-TS	COPD-BS
Characteristics			
Age (years)	67.26 ± 7.32	6.62 ± 7.79^**^	73.77 ± 7.46^**^/^**^
Height (cm)	147 ± 5.6	155.14 ± 7.92^**^/^**^	146.24 ± 5.28/^**^
Weight (kg)	58 ± 11	63.49 ± 13.52^*^	57.78 ± 11.68
BMI (kg/m^2^)	26 ± 6	26.52 ± 6.13	27.5 ± 5.28
Exposure and physiological characteristics			
Tobacco index (pack-years)	0	37.28 ± 11.43	0
Biomass index (h-years of exposure)	0	0	361 ± 177
Exposure to biomass smoke (h-days)	0	0	7.17 ± 2.56
FEV_1_% pred	96.91 ± 13.36	52.40 ± 19.92^**^*	58.40 ± 14.80*
FVC% pred	109.8 ± 9.1	79.49 ± 20.86*	83.10 ± 16.62*
FEV_1_/FVC ratio	92 ± 9.6	53.38 ± 12.62***	56.87 ± 12.11*
BODE	0	3.45 ± 2.86	2.91 ± 1.94
6MWT (m)	0	286.47 ± 174.23	318.91 ± 102.99
Clinical parameters			
PaO_2_ (mm/Hg)	0	55.18 ± 14.65	55.1 ± 1.94
PaCO_2_ (mm/Hg)	0	40.88 ± 11.83	33.95 ± 5.62
SaO_2_ (%)	0	89.54 ± 4.19	89.16 ± 4.03
Glucose	95.0 ± 10.0	101.86 ± 24.5	109.94 ± 28.49**
C-reactive protein	0.28 ± 0.32	0.56 ± 0.62**	0.50 ± 0.51**
GOLD grades Case number (%)			
I	0	5 (8.57)	7 (10)
II	0	27 (38.57)	41 (58.57)
III	0	23 (32.86)	16 (22.86)
IV	0	15 (21.43)	6 (8.57)

Data are expressed as mean ± SD. Definition of abbreviations: BMI, body mass index; FEV_1_% pred, forced expiratory volume in the 1st sec (% predicted); FVC% pred, forced vital capacity (% predicted). Data were analyzed by one-way ANOVA and Tukey’s post hoc test. * P < 0.0001. ** P < 0.01. *** P < 0.05. * vs Control; /* COPD-BS vs COPD-TS

*Abbreviations*: *BMI* body mass index, *COPD-BS* COPD by biomass smoke exposure, *COPD-TS* COPD secondary to tobacco smoking, *FEV*_*1*_*% pred* forced expiratory volume in the 1st sec (% predicted), *FVC% pred* forced vital capacity (% predicted)

### Serum concentration of adipokines, incretins and peptide hormones

**Fig. 1:**
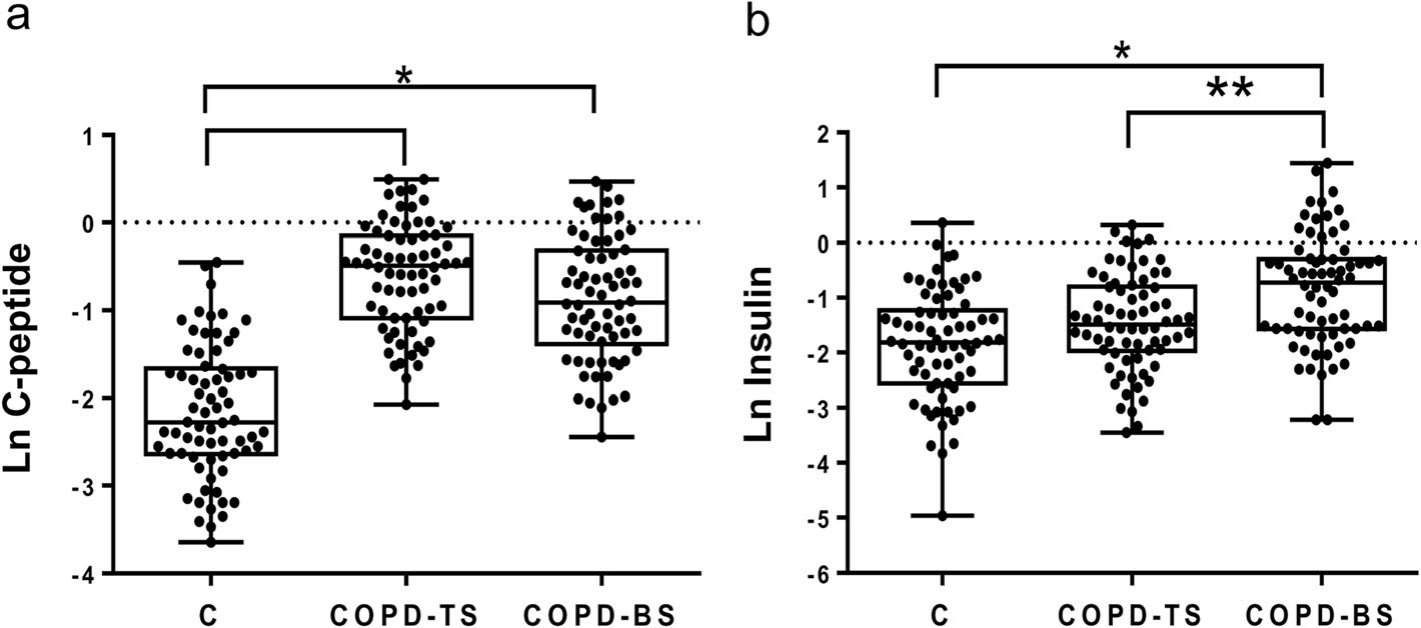
Serum C-peptide and insulin were higher in women with COPD compared with healthy control women (C); but also were greater in COPD-BS related COPD-TS. Data are expressed as natural log (Ln) of ng/ml of C-peptide (**a**), and insulin (**b**) in serum. Kruskal-Wallis follow by Dunn’s post hoc test using the GraphPad Prism (v 6.1 GraphPad Software, Inc., San Diego, CA, USA). * P < 0.0001. ** P < 0.01

**Fig. 2:**
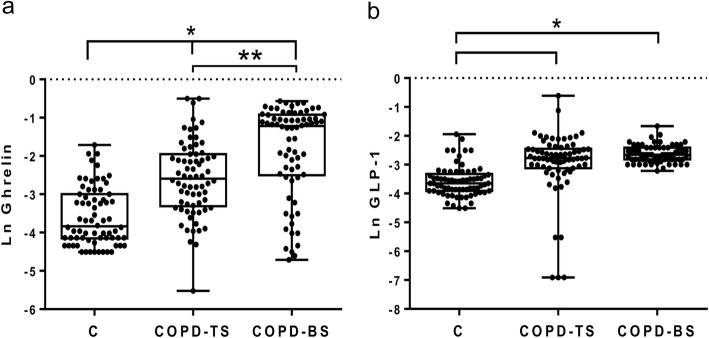
Serum levels of ghrelin and the incretin GLP-1 were greater in women with COPD related to healthy control; ghrelin was also higher in COPD-BS than COPD-TS. Data are expressed as natural log (Ln) of ng/ml of ghrelin (**a**), and incretin GLP-1 (**b**) in serum. Kruskal-Wallis follow by Dunn’s post hoc test using the GraphPad Prism (v 6.1 GraphPad Software, Inc., San Diego, CA, USA). * P < 0.0001. ** P < 0.01

**Fig. 3:**
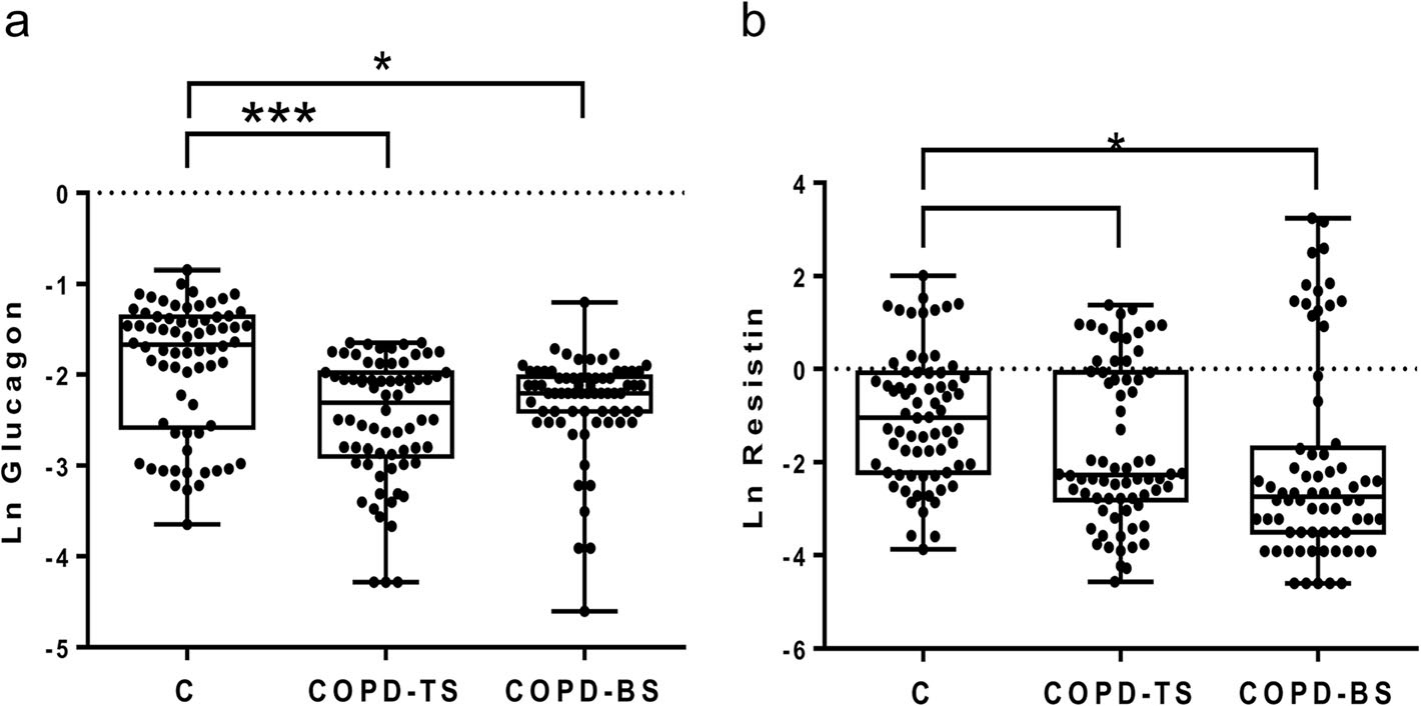
Serum levels of glucagon and resistin were decremented in women with COPD related to healthy control women. Data are expressed as natural log (Ln) of ng/ml glucagon (**a**), and resistin (**b**) in serum. Kruskal-Wallis follow by Dunn’s post hoc test using the GraphPad Prism (v 6.1 GraphPad Software, Inc., San Diego, CA, USA). * P < 0.0001. *** P < 0.05

**Fig. 4:**
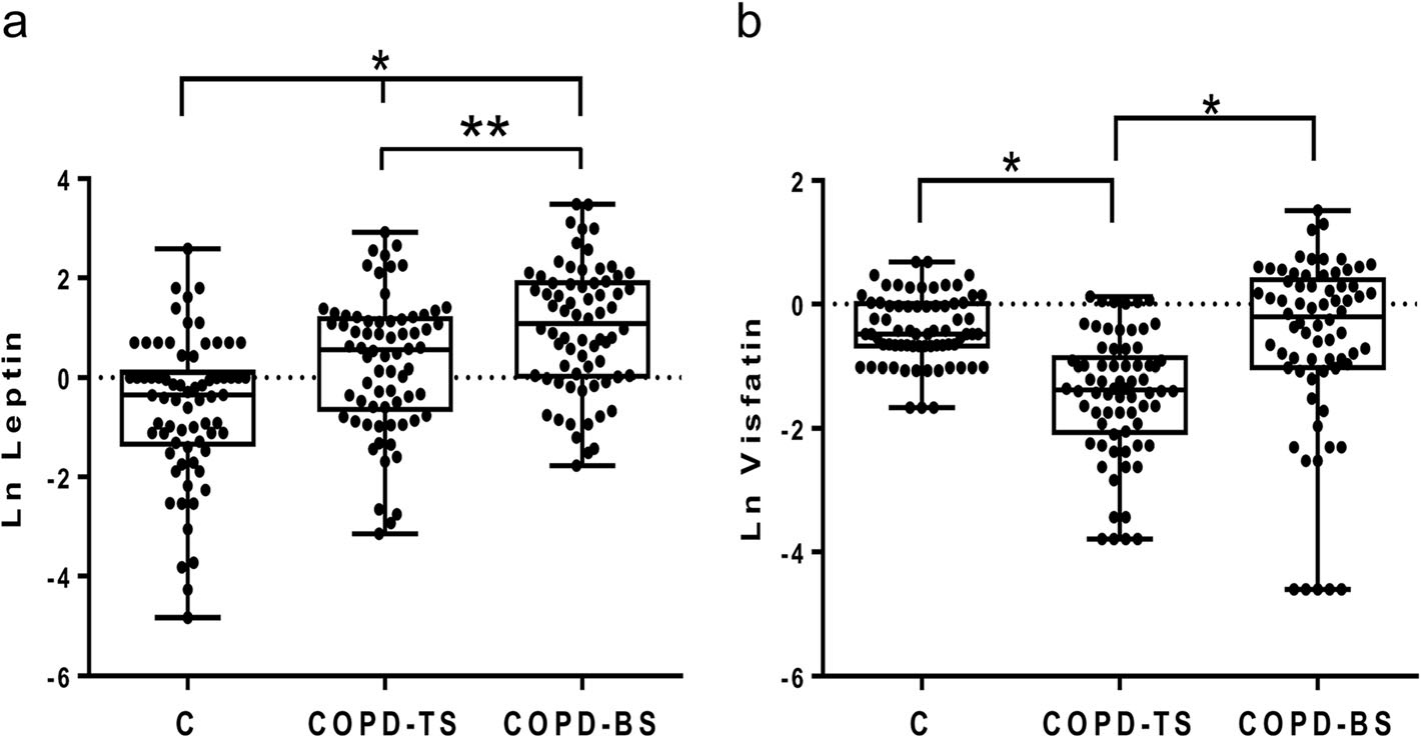
Leptin incremented in serum of COPD-TS and COPD-BS, while visfatin decremented in women with COPD-BS relatively to control women. Data are expressed as natural log (Ln) of ng/ml glucagon (**a**), and resistin (**b**) in serum. Kruskal-Wallis follow by Dunn’s post hoc test using the the GraphPad Prism (v 6.1 GraphPad Software, Inc., San Diego, CA, USA). * P < 0.0001. ** P < 0.01

**Fig. 5:**
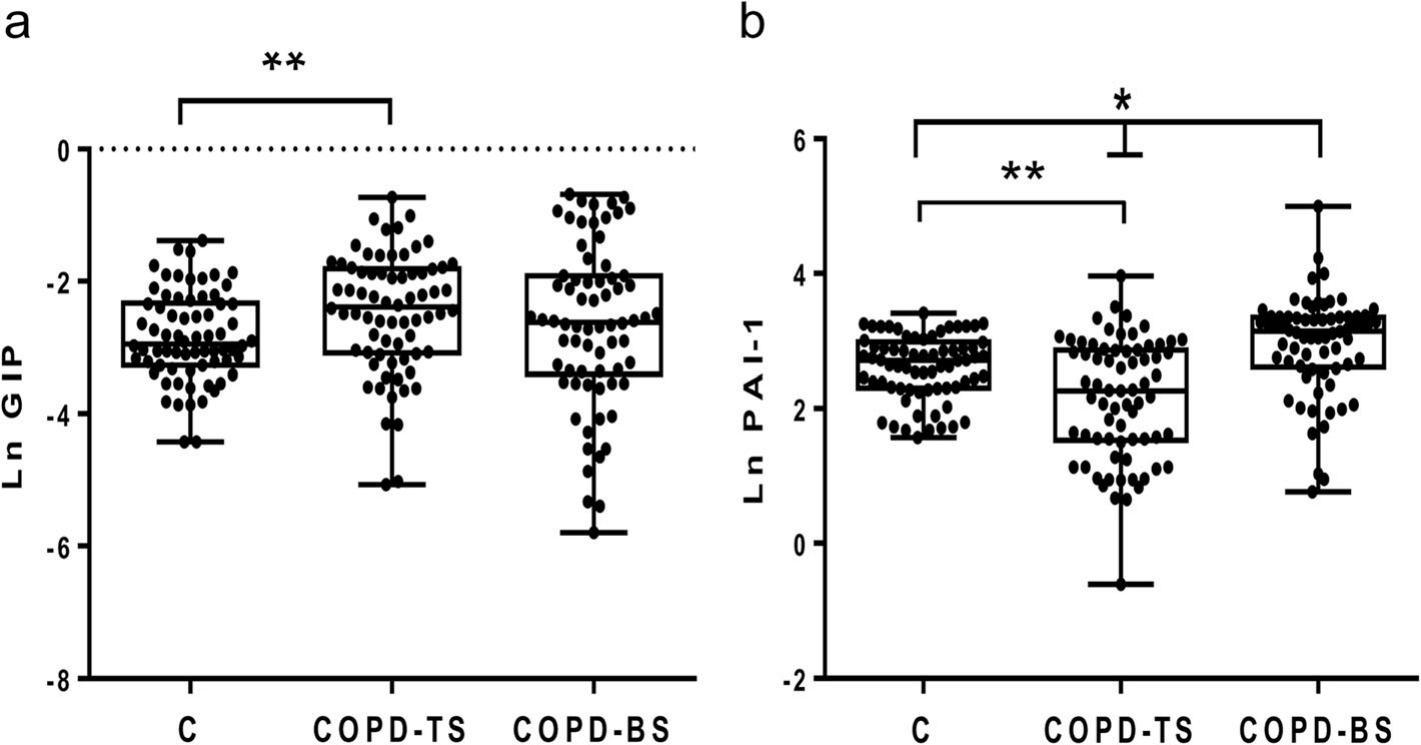
The incretin GIP augmented in serum of COPD-TS, while PAI-1 in both COPD-TS and COPD-BS compared with healthy control women. PAI-1 augmented in COPD-BS respect to COPD-TS. Data are expressed as natural log (Ln) of ng/ml GIP (**a**) and PAI-1 (**b**) in serum. Kruskal-Wallis follow by Dunn’s post hoc test using the GraphPad Prism (v 6.1 GraphPad Software, Inc., San Diego, CA, USA). * P < 0.0001. ** P < 0.01

Table 2 presents median (interquartile ranges) for inflammatory mediators for COPD-BS, COPD-TS and controls. Compared to controls, patients with COPD had higher C-peptide, ghrelin, GLP-1 and leptin, and lower glucagon and resistin; for PAI-1 and visfatin COPD-TS had lower and COPD-BS higher concentrations than controls. Patients with COPD-BS had higher ghrelin, insulin, leptin, PAI-1, and visfatin than patients with COPD-TS (Table 2). Comparative data of serum concentration of inflammatory mediators expressed as natural log (Ln) of ng/ml, between groups of study are shown in Figs. 1, 2, 3, 4 and 5.

**Table 2:** Serum concentration of inflammatory mediators associated with T2DM in women with and without COPD by TS, BS and healthy control women. *n* = 70

Inflammatory mediator	Control	COPD-TS	COPD-BS
C-peptide	0.103 (0.072–0.190)	0.614 (0.333–0.864)*	0.400 (0.251–0.730)*
Ghrelin	0.026 (0.016–0.560)	0.066 (0.035–0.123)*	0.296 (0.082–0.397)*/*
GIP	0.053 (0.038–0.097)	0.092 (0.046–0.164)***	0.073 (0.060–0.090)
GLP-1	0.026 (0.020–0.036)	0.062 (0.044–0.086)*	0.070 (0.060–0.090)*
Glucagon	0.189 (0.076–0.256)	0.100 (0.055–0.139)*	0.110 (0.090–0.133)*
Insulin	0.163 (0.077–0.294)	0.226 (0.140–0.452)	0.485 (0.208–0.743)*/**
Leptin	0.706 (0.264–1.131)	1.737 (0.524–3.239)**	2.96 (1.033–6.720)*/***
PAI-1	15.080 (10.01–19.71)	9.628 (4.626–17.57)***	23.24 (13.64–28.56)*/*
Resistin	0.352 (0.140–0.506)	0.103 (0.207–0.680)***	0.065 (0.097–0.748)*
Visfatin	0.617 (0.508–1.012)	0.253 (0.127–0.423)*	0.817 (0.360–1.490) /*

All measures are expressed in ng/ml. Serum concentration of biomarkers are presented as median (interquartile ranges). * P < 0.0001. ** P < 0.01. *** P < 0.05. * vs control; /* COPD-BS vs COPD-TS

*Abbreviations*: *BS* biomass smoke exposure, *GIP* gastric inhibitory polypeptide, *GLP-1* glucagon-like peptide-1, *PAI-1* plasminogen activator inhibitor-1, *TS* tobacco smoking, *T2DM* type 2 Diabetes mellitus

### Multivariate analysis

Table 3 presents the results of the multivariate analysis of serum concentration of adipokines, incretins and peptide hormones, and the source of exposure of COPD; adjusted for age, BMI, FEV_1_% pred, and metabolic syndrome (MS). Analysis shows that higher concentrations of ghrelin, insulin, PAI-1 have association with BS exposure (Table 3; *P* < 0.05).

**Table 3:** Analysis of variable associated to COPD by BS. Age, BMI, MS, and FEV1% pred were the independent variables

	Ghrelin*	GIP	GLP-1	Glucagon	Insulin*	Leptin	PAI-1*	Resistin	Visfatin*
BS/TS	−0.6366	0.485	0.205	−0.171	−0.541	− 0.334	−0.67	− 0.222	−1.615
	(− 1.11- -0.154)	(− 0.081–1.052)	(− 0.388 - 0.799)	(−0.495–0.152)	(− 0.958 – − 0.123)	(−0.903–0.235)	(−1.020 – − 0.321)	(−1.00–0.555)	(−2.51 – − 0.713)
Age (years)	0.0032	0.027	0.014	0.013	−0.012	− 0.014	−0.027	0.344	0.007
	(−0.235–0.030)	(− 0.003–0.059)	(− 0.018 - 0.047)	(−0.004–0.031)	(− 0.035-0.010)	(−0.046–0.172)	(−0.047 – − 0.008)	(−0.008–0.077)	(−0.042–0.058
BMI	−0.006	− 0.01	−0.016	− 0.011	0.014	0.035	0.001	−0.017	−0.007
	(−0.44–0.032)	(−0.005–0.034)	(− 0.064–0.030)	(− 0.037-0.014)	(−0.019–0.047)	(−0.009–0.080)	(− 0.026 - 0.028)	(−0.079–0.044)	(−0.791–0.064)
MS	− 0.089	−0.022	− 0.321	0.0004	0.014	0.479	0.01	−0.458	−0.576
	(−0.514–0.336)	(−0.557–0.477)	(− 0.845–0.202)	(−0.289–0.280)	(−0.353–0.382)	(−0.022–0.981)	(−0.297–0.318)	(−1.144–0.228)	(−1.372–0.219)
FEV_1_%p	−0.006	0.001	0	0	−0.001	0.001	0.005	−0.013	0
	(−0.018–0.005)	(−0.012–0.015)	(− 0.014 - 0.149)	(−0.007–0.008)	(− 0.011 - 0.009)	(−0.012–0.015)	(−0.003–0.013)	(−0.032–0.006)	(−0.023–0.021)

Multiple comparisons analysis was perform by the statistical software XLSTAT 2016.5 Addinsoft. *P < 0.05

*Abbreviations*: *BMI* Body mass index, *GIP* gastric inhibitory polypeptide, *GLP-1* glucagon-like peptide-1, *MS* Metabolic syndrome, *PAI-1* plasminogen activator inhibitor-1, *TS* tobacco smoking

## Discussion

The main finding of this study is that women with COPD-BS compared with COPD-TS have a distinct serum profile of systemic inflammatory mediators associated with of T2DM. Our analysis was restricted to women because women in Mexico tend to be more exposed to BS than men because them prepared meals, as a domestic role; therefore is very hard for men to develop COPD for this cause. COPD women have an inflammation profile that is higher to the normal controls, as is evidenced by higher plasma CRP; additionally, COPD-BS higher glucose than COPD-TS and controls; which can be accompanied by several inflammatory mediators, such as adipokines, incretins, and peptide hormones. This is dates back up the, multivariate analysis which showed an association of ghrelin, insulin, leptin, PAI-1, and visfatin with the exposure to BS; which support the fact about COPD-BS and COPD-TS could represent different phenotypes of COPD [3, 4]. It is important to note that in this study most of our patients are hypoventilators and hypoxemic, all women showed a FEV1% pred due to the height of Mexico City (2200 m above sea level).

Considering that the BMI showed no difference between the groups of COPD, it might evidence that BMI in not determinant to the develop of systemic inflammation in COPD-BS; thus, the role of ghrelin, insulin, leptin, PAI-1, and visfatin in women exposed to BS seems take an important physiopathogenic role in the develop of systemic inflammation in COPD in this women.

Women with COPD-BS experimented hyperinsulinemia, and potentially an increased risk of developing metabolic complications, such as MS and T2DM [9, 13], playing a crucial role in the reduction of lung function as has been observed in patients presenting COPD and diabetes [17]. Like this, the regulation of glycaemia might be associated with the increment of several of the metabolic and inflammatory biomarkers studies here; and related with the hypertrophic adipose tissue, which function as an endocrine organ hypersecretor of adipokines [3, 4, 13, 18]. Additionally, in states of hyperinsulinemia and hyperglycemia is favored the risk of progression to cardiovascular disease, atherogenesis, vascular endothelial dysfunction and skeletal muscle weakness, in patients with T2DM and COPD-TS [19, 20]. Condition which hyperinsulinemia may contribute to vascular dysfunction in patients with COPD, maybe as part of the prevalence of systemic inflammation, such as have been documented in patients with COPD-TS [21, 22]. Thus, this risk of maintaining the systemic inflammation and develop cardiovascular diseases, seems be more significant in women with COPD-BS than COPD-TS, especially since were C-peptide and insulin are higher in women with COPD-BS. The respiratory epithelia may be affect by the hyperinsulinemia and hyperglycemia, but especially in people with T2DM; where hyperglycemia spend a significant proportion of each day with glucose in their airways secretions, supporting the hyperinsulinemia and insulin resistance [23, 24].

The Framingham Heart Study documented an association between glycemic state and reduced lung function [25]; thus, as was cited previously high glucose concentrations, can lead to an enhanced responsiveness of human airway smooth muscle, but especially in those which the respiratory diseases and T2DM are presented altogether [17]. In a study was shown that plasma glucose levels 2 h after oral administration of glucose (75 g) was inversely related to FEV_1_% pred [26], supporting thus the fact about women with COPD-BS have major risk to affectation in airways than lung parenchyma.

On the other hand, high levels of adiponectin and ghrelin may have an anti-inflammatory effect, such as have been reported that in male with low BMI with severe-to-very severe COPD-TS; however, when BMI is higher the increment in ghrelin seems not be enough to sustenance an anti-inflammatory state, that might contrarested the proinflammatory effect of insulin [27].

Our data also support the role of visfatin as an adipokine that favors the prevalence of systemic inflammation, impairing in the pulmonary diffusing capacity, and decline in the pulmonary function; which was higher in COPD-BS women, and might be responsible, in part, of the systemic inflammatory effect induced by visceral adipose tissue, that is the more pernicious subcutaneous adipose tissue. Visfatin was reported higher in plasma of COPD-TS, associated as a proinflammatory molecule in the pathogenesis of emphysematous COPD, and negatively correlates with pulmonary diffusing capacity [28].

The increase in leptin in COPD patients was previously reported for TS exposure. Leptin is known as a proinflammatory adipokine, secreted by the white adipose tissue, and considered crucial for the development of hypertension in obesity. Which sustenance the fact of the higher risk in COPD-BS women, whom might have a major risk of develop T2DM and hypertension [29].

Similar to the NHANES III study, serum leptin did not show any with COPD-TS with different severities [30]. Other studies suggest that leptin plays a role in respiratory immune responses and inflammation in COPD-TS, even though there was not difference among patients with COPD-TS and controls [31]. Additionally, serum leptin increment was significantly associated with systemic inflammation during phase of exacerbation, and higher levels of leptin (and IL-6) in lungs and serum were associated with the severity of COPD-TS in patients and in rat models [31, 32].

Leptin plays a key role in the regulation of energy homeostasis; increasing evidence suggests that it is also critical for glycemic control, acting as a potential mediator to normalize glycaemia in states of uncontrolled insulin-deficient diabetes [33]. However in women with COPD-BS were observed higher levels of insulin and leptin, which seems not be enough to diminish hyperinsulinemia and hyperglycemia, which the high risk of develop T2DM and other pathogenic effects is maintained [34].

Related to PAI-1, it is relevant indicate that our data confirm the presence of high serum levels of this protein in COPD-TS compared controls [35]. Nevertheless, we found higher levels of PAI-1 in COPD-BS compared with COPD-TS and control women, which mark an important difference in the pathogenic mechanism of COPD, due to PAI-1 is involved in fibrinolysis and turnover of all the molecules of extracellular matrix (ECM). PAI-1 is the chief inhibitor of fibrinolysis that has been strongly associated with thrombosis, obesity, dyslipidemia, insulin resistance, and premature aging, which are parallel circumstances in COPD, but also with the extra-pulmonary manifestations in COPD [36].

In a study of COPD-TS including patients of both gender, PAI-1 was significantly increased, exhibiting an important role in inflammation, ECM turnover, TIMP-1 activity; but showing an inverse correlation with FEV_1_% pred and, FEV_1_/FVC ratio, and a positive correlation with CRP. Additionally, with a multivariable linear study showed that FEV_1_/FVC, CRP, and TIMP-1 were independent parameters associated with PAI-1 [35]; therefore, the increment of serum concentration of PAI-1 is critical to COPD-TS pathogenesis. Also in COPD-BS, where were registered higher serum level than COPD-TS, as other fact distinctive in COPS-BS.

It is relevant indicate that the severity and systemic manifestations of COPD, could be influence by many factors, including genetic factors, characteristics of exposure to TS or BS, personal habits, and socioeconomic status. Thus, to determine the possible causes of the increment of the peptide hormones, adipokines, and incretins, and consequently, the possible develop of T2DM in COPD; multivariable analysis was assessed to know if it really was the exposure to biomass responsible for the changes observed in petidic hormones, adipokines and incretins, the variables were: FEV_1_% pred, age (years), BMI, and biomarkers of MS and T2DM.

Revealing that only ghrelin, insulin, PAI-1, and visfatin displayed an association with the BS exposure; which is relevant because these molecules participate in the glycemic status, and also in the maintaining of hyperinsulinemia; supporting the fact of confer a high risk develop T2DM in those women with COPD-BS.

Related to gender susceptibility to develop COPD, it has been reported that women are more susceptible to smoking effects than men [37]. In this way, several biological differences relative to gender difference have been revised recently [38]; including socio-economic status, genetic profile associated with X chromosome, and history of exposure as a domestic role at home. Thus, our results support the presence of a different phenotype of COPD-BS women relative to COPD-TS, exhibiting a hyperglycemia, hyperinsulinemia, and consequently a major risk of develop T2DM, and the effect that will probable this comorbidity involves.

The dysregulation of adipokines, incretins and peptide hormones, sustenance the fact that prevalence of systemic and pulmonary inflammation represent a major factor to de develop of COPD and eventually the presence of T2DM, and this in both types of COPD [10, 35].

The study has several limitations that have to be taken into account. Pairing by FEV1%p and level of airflow obstruction was successful. Adjusting multivariate analysis by age, BMI, MS, and FEV_1_% pred with levels of inflammatory mediators in COPD women with COPD-BS, may not completely indicate the only the responsible factors of the differences in inflammation and hyperinsulinemia, which also may be still due to differences in body composition. Results support our hypothesized about women with COPD-BS have different biomarkers of T2DM compared with COPD-TS, especially ghrelin, insulin, PAI-1, and visfatin, which were associated with the exposure to BS.

Our data have a similitude with the study of Ho TW and cols (9), whom strongly demonstrated in a wide cohort of Male gender patients with COPD presenting diverse comorbidities, that T2DM was the prevalent comorbidity when COPD was diagnostic, suggesting that targeted surveillance and management of DM are important in clinical care of the COPD population; accordingly, our data support the fact about women exposed to BS also showed T2DM as an important comorbidity.

## Conclusions

Findings showed that women with COPD-BS have distinct profile of adipokInes, incretins, and peptide hormones, with hyperinsulinemia and hyperglycemia, compared with women with COPD-TS, which might confer a major risk of develop T2DM in these women, being it supported especially by the increment in ghrelin, insulin, PAI-1, and visfatin, which were associated with the exposure to BS.

## Abbreviations

BMI: Body mass index
BMI: body mass index
BS: biomass smoke exposure
BS: biomass smoke exposure
COPD: Chronic obstructive pulmonary disease
COPD-BS: COPD associated with biomass smoke exposure
COPD-TS: COPD associated with tobacco smoking
CRP: C-reactive protein
FEV_1_% pred: forced expiratory volume in the 1st sec (% predicted)
FVC% pred: forced vital capacity (% predicted)
GIP: gastric inhibitory polypeptide
GIP: gastric inhibitory polypeptide
GLP-1: glucagon-like peptide-1
GLP-1: glucagon-like peptide-1
MS: Metabolic syndrome
PAI-1: plasminogen activator inhibitor-1
T2DM: type 2 diabetes mellitus
TS: tobacco smoking
